# Effects of Digestible Arginine Supplementation in Low‐Crude‐Protein Diets on Productive Performance, Egg Quality Traits and Immune Response in Breeding Japanese Quails

**DOI:** 10.1002/vms3.71086

**Published:** 2026-07-15

**Authors:** Fatemeh Mortazavi, Ahmad Hassanabadi, Heydar Zarghi

**Affiliations:** ^1^ Department of Animal Science Faculty of Agriculture Ferdowsi University of Mashhad Mashhad Iran

**Keywords:** arginine, egg quality, immune response, Japanese quail, low‐crude‐protein diet, performance

## Abstract

**Background:**

Dietary crude protein is one of the most expensive components of poultry diets. Arginine, an essential amino acid, plays key roles in metabolism, immunity and reproduction.

**Objectives:**

This study evaluated the effects of digestible arginine (dArg) supplementation in a low‐crude‐protein diet on productive performance, egg quality traits, blood metabolites and immune response in breeding Japanese quails.

**Methods:**

A total of 450 ten‐week‐old Japanese quails were allocated to a completely randomized design with six dietary treatments and five replicates per treatment (12 females and 3 males per replicate). A low‐crude‐protein basal diet (16.5% CP) deficient in arginine was supplemented with graded levels of L‐arginine to achieve dietary dArg levels of 1.11%, 1.16%, 1.21%, 1.26%, 1.31% and 1.36%.

**Results:**

Dietary dArg up to 1.36% did not affect egg production, egg weight, feed intake, or feed conversion ratio (*p* > 0.05). However, body weight gain increased linearly (*p* < 0.05), with the highest value observed at 1.36% dArg. Haugh unit showed a positive linear trend (*p* < 0.05), suggesting a potential improvement in albumen quality, while other egg quality traits remained unchanged. Immune response, blood metabolites, fertility and hatchability were not affected by dietary treatments.

**Conclusions:**

A concentration of 1.36% digestible arginine in low‐crude‐protein diets improved body weight gain and albumen quality without affecting overall productive performance, reproductive traits or immune response in breeding Japanese quail.

## Graphical abstract

Increasing digestible arginine up to 1.36% in low‐protein (16.5% CP) diets for breeding Japanese quails improved body weight gain and Haugh unit without affecting egg production, fertility, hatchability, immune response or blood metabolites.

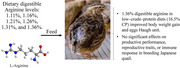

## 1 Introduction

Dietary protein is one of the most costly components of poultry feed, and reducing crude protein (CP) levels without compromising productivity remains a major challenge in poultry nutrition (Lima et al. 2016). Arginine, an essential amino acid for birds, plays critical roles in metabolism, immune function and reproduction. Excess dietary protein not only increases feed costs but also leads to greater nitrogen excretion and environmental pollution (Vieira and Angel 2012). A promising strategy is to reduce CP levels by partially replacing intact protein with crystalline amino acids, which can mitigate the negative effects of low‐CP diets while improving performance and reducing nitrogen output (Belloir et al. 2017; Allameh and Toghyani 2019).

Unlike mammals, poultry cannot synthesize arginine endogenously due to the lack of a functional urea cycle (Corzo et al. 2003). Therefore, they have a relatively high arginine requirement for growth and protein deposition. Arginine serves as a precursor for ornithine and nitric oxide (NO), which are essential for immune regulation, cell division and hormone secretion (Basiouni et al. 2006). However, maintaining a proper arginine‐to‐lysine (Arg:Lys) balance is critical, as antagonism between these two amino acids can impair metabolism (Liaqat et al. 2025). Evidence indicates that supplementing low‐CP diets with crystalline amino acids, including arginine, can reduce production costs and nitrogen emissions without compromising bird performance (Wen et al. 2017; Taheri and Alvani 2020). However, the optimal dietary dArg concentration in breeding Japanese quails is still unclear. While some studies have reported improvements in reproductive performance, egg quality and immune function with arginine supplementation (Kalvandi et al. 2022; Bülbül et al. 2015; Kazemi‐Fard et al. 2017), others have shown limited or context‐dependent effects, particularly when amino acid balance is adequate (Alagawany et al. 2014; Khajali and Wideman 2010; Lima et al. 2022).

To address this research gap, the present study introduced three novel elements: (1) it is the first to determine the optimal dArg level in a low‐CP (16.5%) diet specifically for breeding Japanese quails; (2) it quantifies the ideal Arg:Lys ratio under protein‐restricted feeding; and (3) it provides the simultaneous assessment of productive performance, egg quality, blood metabolites and humoral immunity in a single trial—a combination not previously reported in this species.

Given the unique nutrient demands of breeding quails and the narrow margins for egg component deposition, it is important to evaluate graded concentrations of dArg in low‐CP diets. The present study was therefore conducted to determine the effects of synthetic arginine supplementation on productive performance, egg quality, blood metabolites and immune response in breeding Japanese quails.

## 2 Materials and Methods

### 2.1 Feedstuff Analysis

Prior to the experiment, CP, total and digestible amino acids of feed ingredients were determined using near‐infrared spectroscopy (Evonik Nutrition and Care GmbH). These values were then applied in a least‐cost formulation model to prepare the experimental diets (Table 1).

**TABLE 1:** Analysed nutrient composition of diet ingredients, as‐fed basis.

Analysed nutrient composition^a^	Corn	Wheat	Soybean meal
Metabolizable energy (kcal/kg)	3363	3213	2536
Crude protein (%)	7.93	12.74	40.94
Calcium (%)	0.01	0.05	0.20
Available phosphorus (%)	0.12	0.10	0.20
Sodium (%)	0.02	0.06	0.04
Digestible methionine (%)	0.17	0.18	0.48
Digestible sulphur amino acid (methionine + cystine) (%)	0.33	0.44	0.94
Digestible lysine (%)	0.20	0.33	2.28
Digestible threonine (%)	0.24	0.33	1.34
Digestible valine (%)	0.35	0.50	1.71
Digestible tryptophan (%)	0.05	0.13	0.49
Digestible arginine (%)	0.32	0.63	2.88
Acid linoleic (%)	1.90	1.00	1.80

*Note*: Analysed ingredient analysis was used to calculate nutrient composition (crude protein, calcium and sodium were measured by the AOAC [2019] methods); metabolizable energy, digestible amino acids and available phosphorus were measured by NIR analysis.

^a^ The determined ingredient analysis was used to calculate nutrient composition.

### 2.2 Birds, Diets and Management

This experiment was conducted with 450 breeding Japanese quails (*Coturnix coturnix japonica*). Birds were raised in a floor pen with wood shavings until 35 days of age and subsequently transferred to laying cages. The galvanized wire cages, with mesh floors and dimensions of 50 × 40 × 40 cm (L × W × H; equivalent to 133 cm^2^/bird), were equipped with a trough feeder and 2 nipple drinkers.

Birds were fed a commercial diet until they reached maximum egg production and egg size at 10 weeks of age, after which the trial commenced. Feed and water were available ad libitum throughout the experimental period. Room temperature was maintained at 21 ± 2°C, and lighting was provided using incandescent lamps on a 16 h light:8 h dark cycle with an intensity of 3.5 W/m^2^ (Omary et al. 2024).

At 10 weeks of age, 450 quails with an average live weight of 338.97 g/bird, egg production of 85.67% and egg mass of 11.62 g/bird/day were allocated to 6 dietary treatments in a completely randomized design. Each treatment included 60 females and 15 males, with 5 replicates of 12 females and 3 males per replicate.

Daily metabolizable energy (ME), CP and digestible lysine (dLys) requirements for maintenance, weight gain and egg mass were calculated using the equations of Rostagno et al. (2011). Essential amino acid requirements were determined using amino acid‐to‐lysine ratios recommended in the Brazilian tables for poultry and swine (Rostagno et al. 2011, 2017).

Dietary nutrient levels were formulated based on the Brazilian tables for poultry and swine (Rostagno et al. 2017), which provide updated requirements for Japanese quail breeders. According to these tables, the digestible threonine and valine requirements are 0.48% and 0.72%, respectively, and the available phosphorus requirement is 0.27%, all of which are met by the basal diet (Table 2).

**TABLE 2:** Ingredients and nutrient composition of basal diet, as‐fed basis.

Ingredient	% of diet
Corn	62.43
Wheat	4.83
Soybean meal (44% CP)	24.04
Limestone	6.14
Dicalcium phosphate	0.76
Common salt	0.27
Vitamin premix^a^	0.25
Mineral premix^b^	0.25
L‐lysine‐HCl	0.36
DL‐methionine	0.34
L‐tryptophan	0.04
L‐ threonine	0.09
L‐valine	0.06
Corn starch	0.14
Determined nutrient composition (%)	
Metabolizable energy (kcal/kg)	2900
Crude protein	16.50
Calcium	2.56
Available phosphorus	0.27
Sodium	0.13
Digestible methionine	0.56
Digestible methionine + cystine	0.80
Digestible lysine	0.97
Digestible threonine	0.49
Digestible valine	0.73
Digestible tryptophan	0.20
Digestible arginine	1.11
Acid linoleic	1.67

^a^ Vitamin premix supplied the followings per kg of diet: Vitamin A (all‐trans‐retinol), 4400 IU; vitamin D3 (cholecalciferol), 1000 IU; vitamin E (a‐tocopherol), 11 IU; vitamin K3 (menadione), 2.33 mg; vitamin B1 (thiamine), 2.97 mg; vitamin B2 (riboflavin), 4.4 mg; vitamin B3 (niacin), 22 mg; vitamin B5 (pantothenic acid), 10 mg; vitamin B6 (pyridoxine), 4.45 mg; vitamin B9 (folic acid), 1.9 mg; vitamin B12 (cyanocobalamin), 0.011 mg; vitamin H2 (biotin), 0.18 mg; choline chloride, 487.5 mg; and antioxidant, 1.0 mg.

^b^ Mineral premix supplied the followings per kg of diet: Zn (zinc oxide), 75 mg; Mn (manganese oxide), 75 mg; Fe (iron sulphate), 75 mg; Cu (copper sulphate), 5 mg; I (ethylene diamine di‐hydroiodide), 0.76 mg; Se (sodium selenite), 0.1 mg; and choline chloride, 474 mg.

The ME, CP and digestible amino acids of dietary ingredients were determined using near‐infrared spectroscopy (Evonik Nutrition and Care GmbH).

A basal diet was formulated based on ingredient composition and estimated feed intake (30 g/bird/day for birds aged 9–10 weeks) to meet the nutritional requirements, with the exception of arginine.

The experimental basal diet was formulated to contain 16.5% CP, which was lower than the conventional CP levels commonly recommended for breeding Japanese quails (approximately 18%–20% CP). Therefore, the diet was considered a low‐CP diet. To maintain amino acid adequacy while reducing dietary CP, crystalline amino acids were supplemented according to ideal amino acid ratios recommended by the Brazilian tables for poultry and swine (Rostagno et al. 2011, 2017).

The basal diet contained 1.11% dArg and was divided into six equal portions. Crystalline L‐arginine (99.5% purity, ME = 3220 kcal/kg; Evonik Degussa GmbH) was added at 0.00%, 0.05%, 0.10%, 0.15%, 0.20% and 0.25% of diet, replacing corn starch as filler. After mixing, six experimental diets were obtained with dArg levels of 1.11%, 1.16%, 1.21%, 1.26%, 1.31% and 1.36%, corresponding to dArg/dLys ratios of 1.15%, 1.20%, 1.25%, 1.30%, 1.35% and 1.40%, respectively (Table 2).

The selected dArg levels (1.11%–1.36%) and Arg:Lys ratios (1.15–1.40) were based on recent dose–response studies (Sousa et al. 2022; Morais et al. 2022) to cover a range from below to above the estimated optimum. These ratios were within the safe range, as Arg:Lys antagonism typically occurs above 1.5–1.6 (Liaqat et al. 2025), and recent studies in Japanese quail have used ratios up to 1.40 without adverse effects (Morais et al. 2022). The basal diet met the lysine requirement (0.97% dLys; Rostagno et al. 2017).

The experiment included a two‐week adaptation phase followed by a 12‐week data collection period, divided into three consecutive 28‐day phases (weeks 11–14, 15–18 and 19–22 of age).

### 2.3 Productive Performance

At the beginning and end of the experiment, all birds within each cage were weighed collectively.

Body weight gain was calculated as the difference between initial and final body weights. Mortality was recorded daily. Feed intake was determined as the difference between the feed offered and feed residues at the end of each 28‐day period, adjusted for mortality.

Egg number and egg weight were recorded daily in each replicate. Egg production (%), egg weight (g), egg mass (g/bird/day) and feed conversion ratio (FCR; g feed/g egg mass) were calculated for each 28‐day period (Omary et al. 2024).

### 2.4 Egg Quality Traits

Two eggs per replicate were randomly collected at the end of each 28‐day period and analysed within 6 h of collection at the Poultry Egg Quality Laboratory. Eggs were weighed using digital scale (0.001 g accuracy, GF 400 model, A&D Weighing). Eggs were weighed while submerged in distilled water to measure specific gravity using the following formula:

$$\begin{array}{ccc} & & \text{Specific gravity}\;({\text{g/cm}}^{3})\\ & & \;=\frac{\text{Weight of egg in air}}{\text{Weight of egg in air}-\text{Weight of egg in water}} \end{array}$$

Egg length and width were measured using a digital calliper (0.05 mm accuracy, Insize model 1116‐150), and egg shape index was calculated as:

$$\begin{array}{c} \mathrm{Shape}\;\mathrm{index}=\left(\mathrm{Small}\;\mathrm{diameter}\;/\;\mathrm{Large}\;\mathrm{diameter}\right)\;\times \;100 \end{array}$$

To measure internal quality traits, the eggs were broken onto a glass plate (35 × 25 cm). The Haugh Unit (HU) was calculated as:

$$\begin{array}{ccc} \mathrm{HU} & = & 100\times \log_{10}\left(\mathrm{Albumen}\;\mathrm{height}\;\mathrm{in}\;\mathrm{mm}+7.57\right)\\ & & -1.7\times {\left(\mathrm{Egg}\;\mathrm{weight}\;\mathrm{in}\;g\right)}^{0.37} \end{array}$$

Using a cloth, adhering albumen residues were removed from the yolk, which was then weighed. Eggshells were washed, dried at room temperature for 48 h and weighed. Albumen weight was calculated by subtracting yolk and shell weights from the total egg weight. Relative weights of egg components (yolk, albumen and shell) were determined by dividing their weights by total egg weight. Shell thickness was measured at three points (air cell, equator and sharp end) using a micrometer (0.001 mm accuracy, Mitutoyo model 293–240), and the average was used for analysis (Alsherify and Hassanabadi 2024).

### 2.5 Egg Composition

At the end of the experiment, 10 eggs per replicate were randomly collected for compositional analysis. For chemical composition analyses, egg yolk and albumen were separated immediately after collection and oven‐dried at 75°C for 24 h until constant weight. The dried samples were then ground and analysed for ether extract in the yolk and CP in yolk and albumen according to AOAC (2019) procedures. All chemical composition data were expressed on a dry matter basis.

### 2.6 Fertility and Hatchability

Fifty eggs per replicate were collected during the final week of the experiment and incubated. After hatching, chicks were counted and weighed, and unhatched eggs were broken to determine fertility and hatchability (Alagawany et al. 2014). Fertility and hatchability were calculated as follows:

$$\mathrm{Fertility}\;\left(\%\right)=\left(\mathrm{Number}\;\mathrm{of}\;\mathrm{fertile}\;\mathrm{eggs}\;/\;\mathrm{Total}\;\mathrm{eggs}\;\mathrm{set}\right)\times 100$$

$$\begin{array}{ccc} & & \mathrm{Hatchability}\left(\%\right)\mathrm{from}\;\mathrm{fertile}\;\mathrm{eggs}\\ & & \;=\left(\mathrm{Number}\;\mathrm{of}\;\mathrm{hatched}\;\mathrm{chicks}\;/\;\mathrm{Number}\;\mathrm{of}\;\mathrm{fertile}\;\mathrm{eggs}\right)\times 100 \end{array}$$

$$\begin{array}{ccc} & & \mathrm{Hatchability}\left(\%\right)\;\mathrm{from}\;\mathrm{total}\;\mathrm{eggs}\;\mathrm{set}\\ & & \;=\left(\mathrm{Number}\;\mathrm{of}\;\mathrm{hatched}\;\mathrm{chicks}\;/\;\mathrm{Total}\;\mathrm{eggs}\;\mathrm{set}\right)\times 100 \end{array}$$

### 2.7 Humoral Immune Response

Humoral immunity was evaluated using haemagglutination antibody titres against sheep red blood cells (SRBC). Two birds were randomly selected from each replicate for SRBC immunization, resulting in a total of 60 birds (2 birds × 5 replicates × 6 treatments) used for immune response evaluation. A 5% SRBC suspension in phosphate buffered saline (PBS) was prepared and stored at 4°C. At weeks 20 and 21, 0.5 mL of suspension was injected into the pectoralis major of two birds per replicate. Blood samples (1 mL) were collected 7 days post‐injection from the wing vein. Serum was separated by centrifugation (3000 × *g*, 10 min, 4°C) and stored at −20°C. Antibody titres were determined by haemagglutination assay and expressed as log_2_ values (Alsherify and Hassanabadi 2024).

### 2.8 Blood Collection and Analysis

At the end of the trial, two birds per replicate were randomly selected for blood sampling from the brachial vein. Blood was collected into non‐heparinized tubes, allowed to clot and centrifuged at 1900 × *g* for 5 min at 4°C (Omary et al. 2024). Serum was analysed for triglycerides, total cholesterol, HDL‐C, LDL‐C, total protein, albumin and uric acid using ParsAzmoon commercial kits and an automated biochemical analyser (Cobas Bio, Roche, Basel).

### 2.9 Statistical Analysis

The experiment followed a completely randomized design with 6 treatments and 5 replicates of 12 females and 3 males each. Data normality was checked using the UNIVARIATE procedure of SAS (version 9.4; SAS Institute 2014). Subsequently, data were analysed using the General Linear Model (GLM) procedure. Treatment means were compared using Tukey's test at a 5% significance level. Linear and quadratic regression analyses were performed to evaluate responses to dietary dArg levels.

## 3 Results

### 3.1 Metabolizable Energy and Nutrient Intake

Different supplementation levels of L‐arginine in low‐CP diets had no significant effect on feed intake, ME intake or CP intake between weeks 11 and 22 (*p* > 0.05). However, daily arginine intake increased significantly in a dose‐dependent manner (*p* = 0.0001). The groups receiving diets containing 1.36% and 1.31% dArg showed the highest intakes (473.4 and 467.9 mg/bird/day, respectively), which were significantly higher than that of the control group (396.2 mg/bird/day) (*p* < 0.05) (Table 3).

**TABLE 3:** Effect of different dietary levels of digestible arginine (dArg) on intakes of metabolizable energy (ME), crude protein (CP) and dArg in breeding Japanese quails.

Treatments	ME intake (kcal/bird/d)	CP intake (g/bird/d)	dArg intake (mg/bird/d)
dArg (% of diet)	11–22 weeks	11–22 weeks	11–22 weeks
1.11	103.25	5.87	396.19^d^
1.16	102.25	5.81	409.00d^c^
1.21	104.19	5.92	434.73^bc^
1.26	103.49	5.89	449.67^ab^
1.31	103.57	5.89	467.86^a^
1.36	100.94	5.74	473.38^a^
SEM	1.77	0.10	7.44
	*p*‐value		
Treatment	0.82	0.82	0.0001
Linear	0.33	0.33	0.17
Quadratic	0.32	0.32	0.28

*Note*: Each treatment consisted of five replicates (*n* = 5 cages), with 15 birds per replicate (12 females and 3 males), resulting in 75 birds per treatment. ^a‐d^means with different letters in each column are significantly different (*p* < 0.05).

Abbreviation: SEM, standard error of the mean.

### 3.2 Productive Performance

The influence of dietary dArg levels was evaluated across three production phases (weeks 11–14, 15–18 and 19–22) as well as the overall period (weeks 11–22) for egg production percentage, daily egg mass (g/bird/day), feed intake (g/bird/day) and FCR. No significant differences were observed across the phases or for the total period (*p* > 0.05). Additionally, no significant linear or quadratic effects of dietary dArg levels were detected on any of the evaluated productive performance variables (Tables 4 and 5).

**TABLE 4:** Effect of different dietary levels of digestible arginine (dArg) on egg production and mass in breeding Japanese quails at different ages.

	Egg production (egg/100 bird/d)				Egg mass (g/bird/d)			
Treatments dArg (% of diet)	11–14 weeks	15–18 weeks	19–22 weeks	11–22 weeks (overall)	11–14 weeks	15–18 weeks	19–22 weeks	11–22 weeks (overall)
1.11	85.51	86.88	85.75	86.05	11.60	11.87	11.71	11.73
1.16	85.47	87.00	84.13	85.54	11.43	11.79	11.40	11.514
1.21	86.54	85.81	82.02	84.79	11.81	11.77	11.19	11.59
1.26	84.92	86.91	85.77	85.87	11.49	11.83	11.74	11.69
1.31	88.61	88.92	86.26	87.93	11.89	11.95	11.60	11.82
1.36	82.96	84.24	81.94	83.05	11.49	11.82	11.44	11.58
SEM	2.68	2.21	1.86	1.85	0.36	0.33	0.28	0.27
	*p*‐value							
Treatment	0.78	0.39	0.59	0.59	0.92	0.99	0.72	0.98
Linear	0.53	0.66	0.58	0.63	0.70	0.91	0.67	0.98
Quadratic	0.47	0.57	0.86	0.53	0.73	0.90	0.67	0.96

*Note*: Each treatment consisted of five replicates (*n* = 5 cages), with 15 birds per replicate (12 females and 3 males), resulting in 75 birds per treatment.

Abbreviation: SEM, standard error of the mean.

**TABLE 5:** Effect of different dietary levels of digestible arginine (dArg) on feed intake and feed conversion ratio in breeding Japanese quails at different ages.

Treatments	Feed intake (g/bird/d)				Feed conversion ratio (g/g)			
dArg (% of diet)	11–14 weeks	15–18 weeks	19–22 weeks	11–22 weeks (overall)	11–14 weeks	15–18 weeks	19–22 weeks	11–22 weeks (overall)
1.11	35.60	35.71	37.24	33.86	3.03	3.05	3.14	2.93
1.16	35.26	35.35	36.66	33.75	3.07	3.10	3.11	2.98
1.21	35.93	35.87	36.25	35.65	3.10	3.20	3.08	3.02
1.26	35.68	36.29	36.55	34.21	3.06	3.09	3.10	3.00
1.31	35.71	34.73	37.47	34.94	3.02	2.99	3.14	2.93
1.36	34.80	33.60	36.59	34.22	3.01	2.94	3.11	3.00
SEM	0.61	1.06	0.53	0.53	0.08	0.09	0.08	0.12
	*p*‐value							
Treatment	0.82	0.54	0.59	0.14	0.97	0.46	0.99	0.99
Linear	0.43	0.39	0.43	0.10	0.57	0.24	0.73	0.68
Quadratic	0.32	0.20	0.44	0.14	0.47	0.11	0.75	0.70

*Note*: Each treatment consisted of five replicates (*n* = 5 cages), with 15 birds per replicate (12 females and 3 males), resulting in 75 birds per treatment.

Abbreviation: SEM, standard error of the mean.

### 3.3 Live Body Weight Changes and Average Egg Weight

Live body weight changes at the end of the trial showed a significant positive trend (*p* < 0.05). The group fed diets containing 1.36% dArg exhibited significantly higher body weight gain compared with the control group (*p* < 0.05). Increasing dietary dArg levels from 1.21% to 1.36% resulted in significantly higher weight gain compared to the control, following a linear trend with no significant quadratic effect (Table 6).

**TABLE 6:** Effect of different dietary levels of digestible arginine (dArg) on live body weight change and egg weight in breeding Japanese quails at different ages.

Treatments	Live body weight (g/bird)			Egg weight (g/egg)			
dArg (% of diet)	11 weeks (Initial)	22 weeks (Final)	Change	11–14 weeks	15–18 weeks	19–22 weeks	11–22 weeks (overall)
1.11	336.67	354.65	17.99^e^	13.69	13.7	13.74	13.71
1.16	337.33	356.07	18.74^de^	13.44	13.62	13.54	13.53
1.21	334.67	354.67	20.00^dc^	13.64	13.69	13.64	13.66
1.26	345.73	367.06	21.33^bc^	13.53	13.60	13.68	13.61
1.31	336.80	359.56	22.76^ab^	13.58	13.54	13.68	13.60
1.36	342.67	366.86	24.19^a^	13.85	14.00	13.96	13.94
SEM	3.990	2.884	0.358	0.10	0.12	0.12	0.11
	*p*‐value						
Treatment	0.08	0.008	0.0001	0.15	0.20	0.29	0.18
Linear	0.50	0.52	0.0042	0.12	0.16	0.19	0.12
Quadratic	0.58	0.45	0.20	0.05	0.08	0.07	0.05

*Note*: Each treatment consisted of five replicates (*n* = 5 cages), with 15 birds per replicate (12 females and 3 males), resulting in 75 birds per treatment.

Abbreviation: SEM, standard error of the mean.

Average egg weight did not differ significantly among treatments during any of the three production phases (weeks 11–14, 15–18 and 19–22) or over the entire experimental period (weeks 11–22) (*p* > 0.05). No linear or quadratic effects of dietary dArg levels on average egg weight were observed (Table 6).

### 3.4 Fertility, Hatchability and Hatchling Weight

Dietary dArg levels for breeding quails had no significant effect on their one‐day‐old chick weight, fertility percentage, hatchability based on total eggs set, or hatchability based on fertile eggs (*p* > 0.05). No linear or quadratic effects were observed (Table 7).

**TABLE 7:** Effect of different levels of digestible arginine (dArg) in the diet of breeding Japanese quails on reproductive performance and survivability of chicks from hatch to 22 days of age.

Treatments	Survivability (%)	Fertility (%)	Hatchability (%)		Chick weight (g/bird)
dArg (% of diet)			Set eggs	Fertile eggs	
1.11	97.33	94.40	87.82	93.29	8.76
1.16	97.33	93.66	85.82	91.82	9.34
1.21	97.33	93.63	76.56	81.55	9.34
1.26	98.66	97.40	87.88	90.25	9.07
1.31	97.33	94.89	79.08	83.53	8.92
1.36	98.66	97.99	79.14	80.72	9.17
SEM	1.54	2.15	3.67	3.71	0.15
	*p*‐value				
Treatment	0.95	0.57	0.13	0.08	0.07
Linear	0.93	0.95	0.44	0.37	0.14
Quadratic	0.92	0.63	0.73	0.83	0.15

*Note*: Each treatment consisted of five replicates (*n* = 5 cages), with 15 birds per replicate (12 females and 3 males), resulting in 75 birds per treatment.

Abbreviation: SEM, standard error of the mean.

### 3.5 Egg Quality Parameters and Egg Composition

Dietary dArg levels did not significantly affect egg shape index, shell weight (corrected for egg weight), or shell thickness in any of the production phases (*p* > 0.05) (Tables 8 and 9).

**TABLE 8:** Effect of different dietary levels of digestible arginine (dArg) on egg Haugh unit and shape index in breeding Japanese quails at different ages.

Treatments	Haugh unit				Shape index (%)			
dArg (% of diet)	11–14 weeks	15–18 weeks	19–22 weeks	11–22 weeks (overall)	11–14 weeks	15–18 weeks	19–22 weeks	11–22 weeks (overall)
1.11	57.81	58.90	56.13^c^	57.61^c^	78.61	79.32	76.23	78.05
1.16	59.46	59.47	58.89^bc^	59.27^bc^	79.51	78.84	77.04	78.46
1.21	59.76	57.22	62.15^ab^	59.84^abc^	77.04	78.56	78.54	78.04
1.26	61.74	57.93	62.15^ab^	60.28^abc^	77.31	78.14	73.69	76.38
1.31	61.34	64.54	63.68^ab^	62.54^ab^	78.29	77.57	77.02	77.63
1.36	60.85	63.67	65.79^a^	63.44^a^	78.83	77.55	78.47	78.28
SEM	1.12	2.16	1.33	0.88	1.04	0.95	2.04	0.91
	*p*‐value							
Treatment	0.17	0.11	0.0005	0.001	0.55	0.73	0.58	0.64
Linear	0.05	0.46	0.02	0.24	0.20	0.54	0.66	0.31
Quadratic	0.18	0.16	0.39	0.71	0.21	0.89	0.58	0.34

*Note*: Each treatment consisted of five replicates (*n* = 5 cages), with 15 birds per replicate (12 females and 3 males), resulting in 75 birds per treatment.

Abbreviation: SEM, standard error of the mean.

^a‐c^ Means with different letters in each column are significantly different from each other.

**TABLE 9:** Effect of different dietary levels of digestible arginine (dArg) on egg specific gravity and egg shell thickness in breeding Japanese quails at different ages.

Treatments	Specific gravity (g/cm^3^)				Egg shell thickness (µm)			
dArg (% of diet)	11–14 weeks	15–18 weeks	19–22 weeks	11–22 weeks (overall)	11–14 weeks	15–18 weeks	19–22 weeks	11–22 weeks (overall)
1.11	1.06	1.06	1.07	1.07	242.2	218.2	228.9	229.8
1.16	1.06	1.06	1.06	1.07	259.5	228.9	207.0	231.8
1.21	1.07	1.07	1.07	1.07	243.1	199.1	243.8	228.6
1.26	1.06	1.07	1.06	1.07	238.8	241.5	369.2	283.2
1.31	1.06	1.07	1.05	1.06	246.5	238.6	233.6	239.6
1.36	1.06	1.07	1.07	1.06	255.8	229.2	223.7	238.3
SEM	0.002	0.002	0.005	0.003	7.19	13.61	57.32	20.17
	*p*‐value							
Treatment	0.16	0.19	0.31	0.92	0.29	0.29	0.41	0.40
Linear	0.08	0.01	0.43	0.97	0.50	0.80	0.24	0.27
Quadratic	0.02	0.03	0.6	0.83	0.42	0.95	0.27	0.35

*Note*: Each treatment consisted of five replicates (*n* = 5 cages), with 15 birds per replicate (12 females and 3 males), resulting in 75 birds per treatment.

Abbreviation: SEM, standard error of the mean.

However, specific gravity showed a positive linear and quadratic trend during the first phase (weeks 11–14) and a positive linear trend during the second phase (weeks 15–18), with values increasing as dietary dArg levels rose from 1.11% (control) to 1.36%. Haugh units were significantly affected by dArg levels (*p* < 0.05); both the overall (weeks 11–22) and third‐phase (weeks 19–22) averages showed a significant positive linear trend from the control to the 1.36% dArg group (Table 9).

Dietary dArg levels did not significantly affect the relative weights of eggshell, yolk and albumen (corrected for egg weight), dry matter percentages of yolk and albumen, yolk lipid content or CP percentages of yolk and albumen (*p* > 0.05) (Table 10).

**TABLE 10:** Effect of different dietary levels of digestible arginine (dArg) on relative weights of egg shell, yolk, albumen and contents in breeding Japanese quails at different ages.

Treatments	Shell relative weight (g/100 g egg)				Yolk			Albumen		Albumen and yolk relative weight (g/100 g egg)			
dArg (% of diet)	11–14 weeks	15–18 weeks	19–22 weeks	11–22 weeks (overall)	Solids	Ether extract, %DM	Crude protein, %DM	Solids	Crude protein, %DM	11–14 weeks	15–18 weeks	19–22 weeks	11–22 weeks (overall)
1.11	7.83	8.09	8.56	8.16	54.13	60.42	16.67	13.39	11.34	92.16	91.01	91.43	91.54
1.16	8.39	8.87	8.57	8.61	54.05	58.54	16.67	14.14	11.46	91.60	91.52	91.42	91.51
1.21	8.33	8.80	9.27	8.80	54.34	58.37	16.46	14.31	11.47	91.66	90.72	90.72	91.04
1.26	7.79	8.94	8.78	8.50	53.46	59.47	16.53	13.15	11.19	92.21	90.81	91.21	91.41
1.31	8.03	9.27	9.23	8.85	53.51	58.85	16.67	14.07	11.28	91.95	90.95	90.76	91.22
1.36	8.14	9.40	8.72	8.75	54.66	60.84	16.54	13.99	11.51	91.86	90.40	91.27	91.18
SEM	0.193	0.351	0.377	0.185	0.87	1.07	0.10	0.92	0.81	0.19	0.56	0.37	0.24
	*p*‐value												
Treatment	0.17	0.17	0.62	0.13	0.91	0.49	0.62	0.93	0.44	0.17	0.82	0.62	0.64
Linear	0.61	0.20	0.22	0.08	0.52	0.11	0.39	0.84	0.28	0.59	0.99	0.22	0.44
Quadratic	0.59	0.59	0.29	0.22	0.50	0.07	0.48	0.89	0.23	0.56	0.76	0.29	0.63

*Note*: Each treatment consisted of five replicates (*n* = 5 cages), with 15 birds per replicate (12 females and 3 males), resulting in 75 birds per treatment.

Abbreviation: SEM, standard error of the mean.

### 3.6 Humoral Immune Response

Dietary dArg levels had no significant effect on antibody titres against SRBC during any of the three production phases or over the entire experimental period (*p* > 0.05). Linear and quadratic trends were also not significant (Table 11).

**TABLE 11:** Effect of different dietary levels of digestible arginine (dArg) on antibody titres responses to sheep red blood cell injection.

Treatments	18 weeks of age (7 days after the first injection)			19 weeks of age (7 days after the second injection)		
dArg (% of diet)	Total antibody	IgG	IgA	Total antibody	IgG	IgA
1.11	2.60	1.40	1.20	3.00	1.40	1.60
1.16	3.40	1.80	1.60	3.00	2.00	1.00
1.21	3.00	1.80	1.20	3.00	1.40	1.60
1.26	3.00	1.60	1.40	3.00	1.80	1.20
1.31	3.20	1.80	1.40	4.00	2.60	1.40
1.36	2.80	1.60	1.20	3.8	1.80	2.00
SEM	0.36	0.31	0.25	0.41	0.30	0.32
	*p*‐value					
Treatment	0.70	0.92	0.84	0.30	0.09	0.34
Linear	0.28	0.40	0.60	0.84	0.39	0.26
Quadratic	0.28	0.43	0.54	0.42	0.64	0.14

*Note*: Each treatment consisted of five replicates (*n* = 5 cages), with 15 birds per replicate (12 females and 3 males), resulting in 75 birds per treatment. Data represent means of log2 of the reciprocal of the last dilution exhibiting agglutination.

Abbreviation: SEM, standard error of the mean.

### 3.7 Blood Metabolites

No significant differences were observed in blood metabolite levels (triglycerides, total cholesterol, HDL‐C, LDL‐C, total protein, albumin and uric acid) among treatment groups (*p* > 0.05) (Table 12).

**TABLE 12:** Effect of different dietary levels of digestible arginine (dArg) on blood serum metabolites in breeding Japanese quail determined at 22 weeks of age.

dArg (% of diet)	TG mg/dL	Cho mg/dL	HDL‐C mg/dL	LDL‐C mg/dL	TP g/dL	Alb g/dL	UA mg/dL
1.11	116.40	225.60	103.40	75.60	4.54	2.22	5.16
1.16	11.96	269.20	105.60	79.20	4.66	2.32	5.24
1.21	116.24	240.60	100.60	72.80	4.66	2.28	5.24
1.26	107.36	253.20	100.60	76.40	4.90	2.12	5.74
1.31	117.92	215.60	97.80	76.00	4.92	2.38	4.92
1.36	132.56	202.60	83.20	52.80	4.66	2.29	5.24
SEM	14.53	21.59	7.02	6.80	0.25	0.12	0.38
	*p*‐value						
Treatment	0.88	0.29	0.30	0.11	0.86	0.75	0.77
Linear	0.49	0.25	0.58	0.27	0.31	0.89	0.56
Quadratic	0.34	0.11	0.23	0.08	0.41	0.80	0.55

*Note*: Each treatment consisted of five replicates (*n* = 5 cages), with 15 birds per replicate (12 females and 3 males), resulting in 75 birds per treatment.

Abbreviations: Alb, albumin; Cho, cholesterol; HDL‐C, high‐density lipoprotein cholesterol; LDL‐C, low‐density lipoprotein cholesterol; SEM, standard error of the mean; TG, triglycerides; TP, total protein; UA, uric acid.

## 4 Discussion

### 4.1 Metabolizable Energy and Nutrient Intake

In this study, arginine supplementation to low‐CP diets of laying quails resulted in a significant increase in daily arginine intake and the dArg:dLys ratio without causing significant changes in feed, ME or CP intake. These findings align with those of Reis et al. (2012), who demonstrated that varying arginine‐to‐lysine ratios had no significant effect on feed intake or production performance in quails, and that a ratio of 1.16 was sufficient for optimal performance. Furthermore, Sousa et al. (2022) showed through modelling that arginine acts as a limiting amino acid, and increased intake is associated with improved egg production and body weight maintenance. They recommended a daily intake of 304 mg arginine for optimal production, which corresponds well with the values observed in the present study. Additionally, Morais et al. (2022) reported that an arginine‐to‐lysine ratio of 1.30 provides the best physiological responses and nitrogen retention, with no further benefits from higher ratios.

Overall, the evidence indicates that arginine supplementation in low‐CP diets for laying quails, without altering feed or ME intake, significantly enhances arginine intake and improves the arginine‐to‐lysine ratio, keeping it within the normal range. This improvement may contribute to enhanced nitrogen utilization efficiency and productive performance.

### 4.2 Productive Performance

Dietary dArg levels had no significant effect on productive performance, feed intake or FCR of laying quails under the experimental conditions. The average feed intake (34.44 g/bird/day) and FCR (2.97) were within the expected range for laying quails. Consistent with these findings, Bülbül et al. (2015) reported no significant differences in egg production, egg weight or FCR among treatment groups.

On the other hand, arginine deficiency in other studies has significantly affected breeder poultry performance, with synthetic arginine supplementation restoring productivity. For instance, de Lima et al. (2022) observed that reducing dietary arginine to 0.361% led to a 44% reduction in feed intake, decreased egg production, lower egg weight, reduced body weight and increased FCR. They also reported significant linear and quadratic trends for arginine levels on these parameters and confirmed strong correlations among them. Similarly, Bülbül et al. (2015) reported a 53 g reduction in body weight over the trial period for birds fed arginine at maintenance levels.

The absence of significant effects in this experiment can be mainly attributed to the adequate arginine level in the control diet, which contained 1.11% arginine, close to the NRC (1994) recommendation. This likely met the arginine requirements for maximum egg production under the experimental conditions. Additionally, the optimal arginine‐to‐lysine ratio for growth and production in quails has been reported to be approximately 0.98–1.14 (Omelian and Pozniakovskiy 2017).

Digestible arginine levels had a quadratic effect on egg production percentage in Silva et al. (2012), with the highest production achieved at 1.26% arginine in the diet. These findings align with other studies showing increased egg production as a function of arginine supplementation and the resulting improved arginine‐to‐lysine ratio. Basiouni et al. (2006) reported increased follicle weight in hens fed diets containing 2.05% arginine compared with 1.54%. This effect may be linked to arginine's stimulatory role in luteinizing hormone (LH) secretion, which directly influences ovarian function and follicle development (Basiouni 2009).

Silva et al. (2012) also demonstrated that egg production increased up to 1.26% arginine and then declined at 1.54%, possibly due to amino acid imbalance at very high arginine levels. Considering the metabolic cost of nitrogen excretion with excess arginine (Corzo et al. 2004), future studies should evaluate the simultaneous optimization of arginine, glycine and methionine in breeder diets.

The average egg weight in the present study was 13.51 ± 0.11 g, showing no significant change (Table 6). Most studies, including this one, indicate that egg weight is not influenced by varying arginine levels, especially when the basal diet meets arginine requirements and birds are at peak laying (Sousa et al. 2022; Morais et al. 2022; Maurício et al. 2018). Some studies, such as Silva et al. (2012), reported a linear increase in egg weight with higher arginine levels, but mainly when diets were arginine‐deficient (Sousa et al. 2022). Within conventional ranges, the arginine‐to‐lysine ratio has little effect on egg weight or quality (Maurício et al. 2018; Morais et al. 2022).

Overall, synthetic arginine supplementation up to 0.25% in low‐CP diets may improve growth and body weight gain in laying quails, although significant increases in egg weight are unlikely unless arginine deficiency exists (Sousa et al. 2022; Lima et al. 2022; Morais et al. 2022).

### 4.3 Live Body Weight Changes

In the present experiment, increasing dietary dArg levels resulted in a significant increase in body weight gain, with the highest gain observed at 1.36% dArg compared with the control. Similarly, Bülbül et al. (2015) found that body weight was significantly increased in female laying quails. de Lima et al. (2022) also reported significant effects of arginine levels on performance parameters, including body weight, in laying quails.

They further observed a 53 g reduction in body weight in birds fed with arginine at maintenance levels (0.361 g/bird/day), highlighting the importance of meeting amino acid requirements.

Overall, synthetic arginine supplementation up to 0.25% in low‐CP diets may improve growth and body weight gain in laying quails (Sousa et al. 2022; Lima et al. 2022; Morais et al. 2022)

### 4.4 Fertility, Hatchability and Hatchling Weights

In the present experiment, reproductive parameters were not significantly affected by different dietary dArg levels.

Optimal reproductive performance requires balanced amino acid ratios in diets along with proper nutritional, environmental and health conditions (Carvalho et al. 2022). Although arginine is vital for maintenance and egg production, excess levels beyond requirements do not improve reproductive performance and may disrupt metabolic balance (Sousa et al. 2022). Silva et al. (2012) similarly reported that increasing arginine levels in broiler breeder diets did not affect hatchability or embryo mortality.

According to previous studies and the current results, fertility, hatchability and embryo survival are unaffected by arginine supplementation in low‐CP diets under normal rearing conditions. Significant effects are more likely when baseline amino acid levels are below maintenance requirements or when birds are exposed to stressors such as heat. Under such conditions, arginine supplementation can mitigate negative effects (Silva et al. 2012; Kalvandi et al. 2022; Karbalaei Babaei et al. 2012).

For example, Kalvandi et al. (2022) reported that increasing dietary arginine in heat‐stressed quails improved hatchability in a linear manner and fertility in a quadratic manner. Mechanistically, arginine's involvement in nitric oxide synthesis and enhanced ovarian blood flow may contribute to improved reproductive performance and egg quality.

### 4.5 Egg Quality Parameters and Egg Composition

Overall, most egg quality indices including shape index, shell weight, shell thickness and yolk and albumen composition were not significantly influenced by dietary dArg levels. However, some traits, particularly HU and egg specific gravity, improved with higher dArg levels.

The HU, a widely accepted measure of albumen quality, was significantly improved by increasing dietary dArg levels, especially during the later production phase (weeks 19–22) and across the overall period. The positive linear response suggests that dArg may enhance albumen quality under reduced CP conditions. These findings are consistent with reports in poultry where arginine supplementation improved protein metabolism and albumen quality, likely through roles in nitric oxide synthesis, hormone regulation and polyamine production. Polyamines (putrescine and spermidine) derived from arginine have been shown to stimulate protein synthesis in the oviduct magnum, potentially enhancing albumen deposition (Yang et al. 2021), although direct evidence in quail remains limited (Kalvandi et al. 2022; Khajali and Wideman 2010). Improved HU in breeder quails is particularly relevant, as albumen quality directly affects hatchability and chick viability.

Although dArg did not significantly affect shell thickness or relative shell weight, specific gravity exhibited positive linear and quadratic trends in the early (weeks 11–14) and mid‐production phases (weeks 15–18). This suggests that arginine may contribute to shell integrity. Arginine's role in calcium metabolism and collagen synthesis, both essential for shell formation, may explain these results. Nonetheless, the lack of significant differences in overall shell weight and thickness implies that beneficial effects may be modest or phase‐dependent. Similar findings have been reported in laying hens, where arginine supplementation improved shell strength under certain conditions but inconsistently across studies (Kazemi‐Fard et al. 2017; Onderci et al. 2006; Khajali and Wideman 2010).

Relative weights of yolk, albumen and shell, as well as yolk and albumen DM, CP and lipid contents, were not significantly influenced by dietary dArg. This suggests that within the tested range, dietary dArg did not markedly alter nutrient partitioning into egg components. These results agree with previous studies in poultry reporting limited effects of arginine on yolk lipid deposition, except under severe deficiency or amino acid imbalance (Kazemi‐Fard et al. 2017; Bülbül et al. 2015; Reis et al. 2012).

### 4.6 Humoral Immune Response

The results indicated no significant effect of varying dietary dArg concentrations on the humoral immune response of laying quails. Neither linear nor quadratic trends were significant. These findings are consistent with reports showing no enhancement in humoral immune responses, including antibody titres against SRBC, due to synthetic L‐arginine supplementation in reduced‐protein diets of laying quails at various production stages (Liaqat et al. 2025; Ognik et al. 2021).

Kalvandi et al. (2022) observed that arginine supplementation significantly increased secondary antibody titres against SRBC under heat stress, but not under thermoneutral conditions. This suggests that arginine's immunomodulatory effects are primarily evident under stress, serving as a potential nutritional strategy to mitigate heat stress. Similarly, Karbalaei Babaei et al. (2012) reported no effects of standard or low‐CP diets on the humoral response to Newcastle and influenza vaccines in broiler breeder hens.

Thus, the absence of significant effects in the present study may be attributed to sufficient baseline arginine levels in the control diet and appropriate arginine‐to‐lysine ratios across treatments, limiting arginine's potential to further modulate immune responses under stable rearing conditions.

### 4.7 Blood Metabolites

Blood metabolites of laying quails were not affected by different levels of arginine supplementation. Consistent with these results, Kazemi‐Fard et al. (2017) reported no significant effects of increased dietary L‐arginine on blood parameters in laying hens.

In contrast, some studies have shown reductions in blood cholesterol and triglycerides with high dietary arginine (Emadi et al. 2010). Arginine, via nitric oxide production, may influence lipid metabolism and lower cholesterol and triglycerides (Khajali and Wideman 2010). Kheiri and Landy (2020) also reported that increasing the arginine‐to‐lysine ratio could reduce uric acid and triglycerides and increase HDL‐C, although effects on other metabolites and growth performance were limited. Additionally, in ovo injection of arginine significantly improved some physiological and production indices, including reductions in cholesterol and triglycerides (Al‐Daraji et al. 2012). However, synthetic amino acid supplementation in mature or breeder birds generally produces milder effects.

Overall, the evidence suggests that dietary L‐arginine supplementation at the tested levels does not significantly impact blood metabolites in Japanese quails.

## 5 Conclusions

Varying levels of digestible arginine in low‐protein diets of breeding Japanese quails significantly increased arginine intake and positively influenced live body weight gain. However, no notable improvements were observed in feed intake, feed conversion ratio, egg production, egg mass, fertility, hatchability or hatchling weight. Egg quality traits were largely unaffected, except for a positive linear trend in Haugh units, indicating improved albumen quality with higher dArg levels suggesting a potential improvement in albumen quality. Furthermore, humoral immune response and blood metabolite profiles showed no significant changes across dietary treatments. These findings suggest that while increased dietary arginine can enhance body weight gain in breeding quails fed low‐protein diets, it does not markedly affect productive performance, reproductive outcomes, or immune function. Optimizing arginine supplementation may therefore offer benefits for growth but has limited impact on other production and health parameters in this species. Further researches should explore long‐term effects and underlying mechanisms to better clarify arginine's role in quail nutrition.

## Author Contributions

**Fatemeh Mortazavi**: writing first draft, investigation, data curation, software. **Ahmad Hassanabadi**: project administration, supervision, software, methodology, conceptualization, funding acquisition, writing – review and editing. **Heydar Zarghi**: advising, review, editing.

## Funding

This research was supported by the Ferdowsi University of Mashhad (Project No. 3/56204).

## Ethics Statement

The authors confirm that the ethical policies of the journal, as noted in the journal's author guidelines page, have been adhered to and the appropriate ethical review committee approval has been received. The authors confirm that they have followed EU standards for the protection of animals used for scientific purposes and feed legislation. Also, this study was conducted according to the procedures established by the Iranian Ministry of Agriculture (Experimental Authorization No. ASRI‐2016‐95014).

## Conflicts of Interest

The authors declare no conflicts of interest.

## Data Availability

The data that support the findings of this study are available from the corresponding author upon reasonable request.

## References

[vms371086-bib-0001] Alagawany, M. , M. E. A. El‐Hack , V. Laudadio , and V. Tufarelli . 2014. “Effect of Low‐Protein Diets With Crystalline Amino Acid Supplementation on Egg Production, Blood Parameters and Nitrogen Balance in Laying Japanese Quails.” Avian Biology Research 7, no. 4: 235–243. 10.3184/175815514X14152945166603.

[vms371086-bib-0002] Al‐Daraji, H. J. , A. A. Al‐Mashadani , W. K. Al‐Mashadani , A. S. Al‐Hassani , and H. A. Mirza . 2012. “Effect of In Ovo Injection With L‐Arginine on Productive and Physiological Traits of Japanese Quail.” South African Journal of Animal Science 42, no. 2: 139–145. 10.4314/sajas.v42i2.6.

[vms371086-bib-0003] Allameh, S. , and M. Toghyani . 2019. “Effect of Dietary Valine Supplementation to Low Protein Diets on Performance, Intestinal Morphology and Immune Responses in Broiler Chickens.” Livestock Science 229: 137–144. 10.1016/j.livsci.2019.09.025.

[vms371086-bib-0004] Alsherify, S. M. , and A. Hassanabadi . 2024. “Effects of Adding Different Levels of Fructooligosaccharide to Diet on Productive Performance, Egg Quality Traits, Immune Response and Blood Metabolites in Commercial Laying Hens.” Veterinary Medicine and Science 10, no. 5: e1550. 39119788 10.1002/vms3.1550 PMC11310662

[vms371086-bib-0005] Association of Official Analytical Chemists . 2019. Official Methods of Analysis. 21st ed. AOAC.

[vms371086-bib-0006] Basiouni, G. , H. Najib , M. M. Zaki , and A. S. Al‐Ankari . 2006. “Influence of Extra Supplementation With Arginine and Lysine on Overall Performance, Ovarian Activities and Humoral Immune Response in Local Saudi Hens.” International Journal of Poultry Science 5, no. 5: 441–448. 10.3923/ijps.2006.441.448.

[vms371086-bib-0007] Basiouni, G. F. 2009. “The Effect of Feeding an Extra Amounts of Arginine to Local Saudi Hens on Luteinizing Hormone Secretion.” Journal of Biological Sciences 9, no. 6: 617–620. http://scialert.net/fulltext/?doi=jbs.2009.617.620.

[vms371086-bib-0008] Belloir, P. , B. Méda , W. Lambert , et al. 2017. “Reducing the CP Content in Broiler Feeds: Impact on Animal Performance, Meat Quality and Nitrogen Utilization.” Animal 11, no. 11: 1881–1889. 10.1017/S1751731117000660. 28462773 PMC5645801

[vms371086-bib-0009] Bülbül, T. , E. Ulutaş , and M. Evcimen . 2015. “Effects of Dietary Supplementation of Arginine and Lysine on Performance and Egg Quality Characteristics of Laying Quails.” Ankara Üniversitesi Veteriner Fakültesi Dergisi 62, no. 4: 307–312. 10.1501/vetfak_0000002697.

[vms371086-bib-0010] Carvalho, L. C. , T. S. A. Mani , M. B. Lima , et al. 2022. “Determination of the Optimal In‐Feed Amino Acid Ratio for Japanese Quail Breeders Based on Utilization Efficiency.” Animals 12, no. 21: 2953. 10.3390/ani12212953. 36359076 PMC9656694

[vms371086-bib-0011] Corzo, A. , M. Kidd , D. Burnham , and B. Kerr . 2004. “Dietary Glycine Needs of Broiler Chicks.” Poultry Science 83, no. 8: 1382–1384. 10.1093/ps/83.8.1382. 15339014

[vms371086-bib-0012] Corzo, A. , E. Moran Jr. , and D. Hoehler . 2003. “Arginine Need of Heavy Broiler Males: Applying the Ideal Protein Concept.” Poultry Science 82, no. 3: 402–407. 10.1093/ps/82.3.402. 12705400

[vms371086-bib-0013] de Lima, M. B. , M. G. B. L. de Sousa , A. R. T. Minussi , et al. 2022. “Arginine Requirement for Egg Production in Japanese Quail.” Poultry Science 101, no. 6: 101841. 10.1016/j.psj.2022.101841. PMC9048065 35462207

[vms371086-bib-0014] Emadi, M. , K. Kaveh , M. H. Bejo , et al. 2010. “Growth Performance and Blood Parameters as Influenced by Different Levels of Dietary Arginine in Broiler Chickens.” Journal of Animal and Veterinary Advances 9, no. 1: 70–74.

[vms371086-bib-0015] Kalvandi, O. , A. Sadeghi , and A. Karimi . 2022. “Arginine Supplementation Improves Reproductive Performance, Antioxidant Status, Immunity and Maternal Antibody Transmission in Breeder Japanese Quail Under Heat Stress Conditions.” Italian Journal of Animal Science 21, no. 1: 8–17. 10.1080/1828051X.2021.2013136. PMC6852882 31807638

[vms371086-bib-0016] Karbalaei Babaei, H. , A. Zamani Moghaddam , and F. Khajeali . 2012. “The Effect of Low‐Protein Diet Supplemented With Amino Acids on Performance, Hatchability, and Immune Response of Broiler Breeder Hens During the Second Production Cycle [in Persian].” Iranian Journal of Animal Science Research 4, no. 2: 109–114. 10.22067/ijasr.v4i2.15135.

[vms371086-bib-0017] Kazemi‐Fard, M. , S. Yousefi , M. Rezaei , S.h. Bahram , and T. Saberi Far . 2017. “The Effect of L‐Arginine Supplementation on Performance, Egg Quality Traits, Blood and Hormonal Parameters in Laying Hens During the Late Production Period.” Research in Animal Production 8, no. 17: 11–17. 10.22059/jap.2025.391217.623837.

[vms371086-bib-0018] Khajali, F. , and R. F. Wideman . 2010. “Dietary Arginine: Metabolic, Environmental, Immunological and Physiological Interrelationships.” World's Poultry Science Journal 66, no. 4: 751–766. 10.1017/S0043933910000711.

[vms371086-bib-0019] Kheiri, F. , and N. Landy . 2020. “Growth Performance, Intestinal Morphology, Serum Biochemical and Hematological Parameters in Japanese Quail (*Coturnix japonica*) Fed Supplemental L‐Arginine.” Brazilian Journal of Poultry Science 22, no. 03: eRBCA‐2019. 10.1590/1806-9061-2019-1200.

[vms371086-bib-0020] Liaqat, W. , U. Anwar , A. Fatima , et al. 2025. “Effect of Ideal Amino Acid Ratio of Arginine to Lysine on Intake, Nutrient Digestibility, Growth Performance, Antibody Titers of Newcastle Disease and Infectious Bronchitis Disease, and Carcass Characteristics of Broilers.” Animals 15, no. 2: 135–147. 10.3390/ani15020135. 39858135 PMC11759158

[vms371086-bib-0021] Lima, H. , M. V. Morais , and I. D. B. Pereira . 2022. “Updates in Research on Quail Nutrition and Feeding: A Review.” World's Poultry Science Journal 79, no. 1: 69–93. 10.1080/00439339.2022.2150926.

[vms371086-bib-0022] Lima, H. J. D. , S. L. T. Barreto , J. L. Donzele , G. S. Souza , R. L. Almeida , and I. F. F. Tinoco . 2016. “Digestible Lysine Requirement for Growing Japanese Quails.” Journal of Applied Poultry Research 25, no. 4: 483–491. 10.3382/japr/pfw030.

[vms371086-bib-0023] Maurício, T. V. , J. G. de Vargas , M. F. de Souza , B. da Costa Ferrreira , R. V. Nunes , and F. M. Vieites . 2018. “Ratio of Digestible Lysine to Arginine in Japanese Laying Quails.” Semina: Ciências Agrárias 39, no. 1: 299–310. 10.5433/1679-0359.2018v39n1p299.

[vms371086-bib-0024] Morais, M. V. M. , H. J. D. A. Lima , F. N. A. Silva , and M. V. F. C. Gomes . 2022. “Performance and Egg Quality of Laying Japanese Quails (*Coturnix coturnix japonica*) Reared in Hot Climate as a Function of Digestible Arginine: Lysine Ratios in the Diet.” Indian Journal of Animal Sciences 92, no. 10: 1216–1221. 10.56093/ijans.v92i10.114248.

[vms371086-bib-0025] NRC . 1994. Nutrient Requirements of Poultry. National Academy Press. 10.17226/2114.

[vms371086-bib-0026] Ognik, K. , D. Mikulski , P. Konieczka , et al. 2021. “The Immune Status, Oxidative and Epigenetic Changes in Tissues of Turkeys Fed Diets With Different Ratios of Arginine and Lysine.” Scientific Reports 11, no. 1: 15975. 10.1038/s41598-021-95529-y. 34354153 PMC8342415

[vms371086-bib-0027] Omary, M. A. , H. Zarghi , and A. Hassanabadi . 2024. “Effects of Dietary Digestible Lysine Levels in Breeding Japanese Quails on Productive and Reproductive Performance, Egg Quality, Blood Metabolites and Immune Responses.” Veterinary Medicine and Science 10, no. 6: e70038. 39367780 10.1002/vms3.70038 PMC11452902

[vms371086-bib-0028] Omelian, A. M. , and Y. V. Pozniakovskiy . 2017. “Arginine and Lysine: The Impact of Their Correlation on the Productivity of Young Quails.” Scientific Messenger of LNU of Veterinary Medicine and Biotechnologies Series: Agricultural sciences 19, no. 74: 44–47. 10.15421/nvlvet7410.

[vms371086-bib-0029] Onderci, M. , N. Sahin , K. Sahin , et al. 2006. “Dietary Arginine Silicate Inositol Complex During the Late Laying Period of Quail at Different Environmental Temperatures.” British Poultry Science 47, no. 2: 209–215. 10.1080/00071660600611052. 16641032

[vms371086-bib-0030] Reis, R. D. S. , S. L. D. T. Barreto , W. D. S. Abjaude , D. R. Dutra , M. Santos , and E. D. Paula . 2012. “Relationship of Arginine With Lysine in Diets for Laying Japanese Quails.” Revista Brasileira de Zootecnia 41: 106–110. 10.1590/S1516-35982012000100016.

[vms371086-bib-0031] Rostagno H. S. , L. F. T. Albino , M. I. Hannas , et al ., eds. 2017. Brazilian Tables for Poultry and Swine: Composition of Feedstuffs and Nutritional Requirements.Viçosa, MG: UF. 4th ed. Federal University of Viçosa.

[vms371086-bib-0032] Rostagno, H. S. , L. F. T. Albino , J. L. Donzele , et al. 2011. Brazilian Tables for Poultry and Swine: Composition of Feedstuffs and Nutritional Requirements. 3rd ed. Universidade Federal de Viçosa.

[vms371086-bib-0033] SAS Institute . 2014. SAS User's Guide, Version 9.4. SAS Institute Inc.

[vms371086-bib-0034] Silva, L. M. G. S. , A. E. Murakami , J. I. M. Fernandes , D. Dalla Rosa , and J. F. Urgnani . 2012. “Effects of Dietary Arginine Supplementation on Broiler Breeder Egg Production and Hatchability.” Brazilian Journal of Poultry Science 14: 267–273. 10.1590/S1516-635X2012000300007.

[vms371086-bib-0035] Sousa, M. , M. Lima , R. B. Vieira , et al. 2022. “Modeling the Response of Japanese Quail to Arginine Intake.” Peer J 10: e14337. 10.7717/peerj.14337. 36573239 PMC9789692

[vms371086-bib-0036] Taheri, H. , and S. Alvani . 2020. “Effect of Protein and Energy‐Reduced or Protein‐Reduced Diet on Mortality and Performance of Broiler Chickens Reared at a High‐Altitude Area.” Poultry Science Journal 8, no. 2: 129–133. 10.22069/psj.2020.17492.1537.

[vms371086-bib-0037] Vieira, S. , and C. Angel . 2012. “Optimizing Broiler Performance Using Different Amino Acid Density Diets: What are the Limits?” Journal of Applied Poultry Research 21, no. 1: 149–155. 10.3382/japr.2011-00476.

[vms371086-bib-0038] Wen, Z. , Y. Du , M. Xie , X. Li , J. Wang , and P. Yang . 2017. “Effects of Low‐Protein Diets on Growth Performance and Carcass Yields of Growing French Meat Quails (France *Coturnix coturnix*).” Poultry Science 96, no. 5: 1364–1369. 10.3382/ps/pew321. 27702918

[vms371086-bib-0039] Yang, H. , B. R. Lee , H. C. Lee , et al. 2021. “Isolation and Characterization of Cultured Chicken Oviduct Epithelial Cells and In Vitro Validation of Constructed Ovalbumin Promoter in These Cells.” Animal Bioscience 34, no. 8: 1321. 10.5713/ab.20.0627. 33332940 PMC8255889

